# Phase II, multi-center, open-label, single-arm clinical trial evaluating the efficacy and safety of Mycophenolate Mofetil in patients with high-grade locally advanced or metastatic osteosarcoma (ESMMO): rationale and design of the ESMMO trial

**DOI:** 10.1186/s12885-020-06751-2

**Published:** 2020-03-30

**Authors:** Nut Koonrungsesomboon, Nuttapong Ngamphaiboon, Natavudh Townamchai, Pimpisa Teeyakasem, Chaiyut Charoentum, Pimlak Charoenkwan, Rungrote Natesirinilkul, Lalita Sathitsamitphong, Touch Ativitavas, Parunya Chaiyawat, Jeerawan Klangjorhor, Suradej Hongeng, Dumnoensun Pruksakorn

**Affiliations:** 1 Department of Pharmacology, Faculty of Medicine, Chiang Mai University, Chiang Mai, Thailand; 2 Muscoloskeletal Science and Translational Research (MSTR) Center, Chiang Mai University, Chiang Mai, Thailand; 3 Department of Internal Medicine, Faculty of Medicine Ramathibodi Hospital, Mahidol University, Nakhon Pathom, Thailand; 4 Department of Medicine, Faculty of Medicine, Chulalongkorn University, Bangkok, Thailand; 5 Department of Internal Medicine, Faculty of Medicine, Chiang Mai University, Chiang Mai, Thailand; 6 Departmnet of Pediatrics, Faculty of Medicine, Chiang Mai University, Chiang Mai, Thailand; 7 Department of Pediatrics, Faculty of Medicine Ramathibodi Hospital, Mahidol University, Nakhon Pathom, Thailand; 8 Department of Orthopedics, Faculty of Medicine, Chiang Mai University, 110 Intawaroros, Sriphoom, Muang, Chiang Mai, 50200 Thailand; 9 Biomedical Engineering Institute, Chiang Mai University, Chiang Mai, Thailand

**Keywords:** Osteosarcoma, Mycophenolate mofetil, Phase II, Clinical trial, Cancer

## Abstract

**Background:**

Clinical outcomes of patients with osteosarcoma remain unsatisfactory, with little improvement in a 5-year overall survival over the past three decades. There is a substantial need for further research and development to identify and develop more efficacious agents/regimens in order to improve clinical outcomes of patients for whom the prognosis is unfavorable. Recently, mycophenolate mofetil, a prodrug of mycophenolic acid, has been found to have anticancer activity against osteosarcoma in both in vitro and animal experiments, so that further investigation in humans is warranted.

**Methods:**

A total of 27 patients with high-grade locally advanced or metastatic osteosarcoma will be enrolled into this phase II, multi-center, open-label, single-arm, two-stage clinical trial. The main objectives of this study are to determine the efficacy and safety of mycophenolate mofetil in the patients. The primary endpoint is progression-free survival at 16 weeks; the secondary endpoints include progression-free survival, overall survival, overall response rate, safety parameters, pharmacokinetic parameters, biomarkers, pain score, and quality of life. Mycophenolate mofetil at the initial dose of 5 g/day or lower will be administered for 4 cycles (28 days/cycle) or until disease progression or unacceptable toxicity. The dose of mycophenolate mofetil may be reduced by 1–2 g/day or withheld for some Grade 3 or Grade 4 toxicities whenever clinically needed. The duration of study participation is approximately 4–5 months, with a minimum of 12 study visits. If mycophenolate mofetil proves beneficial to some patients, as evidenced by stable disease or partial response at 16 weeks, administration of mycophenolate mofetil will continue in the extension period.

**Discussion:**

This trial is the first step in the translation of therapeutic potential of mycophenolate mofetil emerging from in vitro and animal studies into the clinical domain. It is designed to assess the efficacy and safety of mycophenolate mofetil in patients with high-grade locally advanced or metastatic osteosarcoma. The results will provide important information about whether or not mycophenolate mofetil is worth further development.

**Trial registration:**

This trial was prospectively registered on Thai Clinical Trials Registry (registration number: TCTR20190701001). The posted information will be updated as needed to reflect protocol amendments and study progress.

## Background

Osteosarcoma is one of the most common primary malignant tumors of the bone in adolescents and young adults, with the incidence of approximately 2–4 per million worldwide [1–4]. The substantial advance in the treatment of osteosarcoma occurs in the 1970s and 1980s, when chemotherapy was shown to significantly improve the survival outcome of the patients with localized disease undergoing surgical resection [5, 6]. However, little further progress has been observed since then, with a 5-year overall survival remaining at approximately 70 and 30% in patients with localized and metastatic disease at diagnosis, respectively [7, 8]. At present, the combination of surgical resection and systemic chemotherapy with, at least, three active drugs (i.e., doxorubicin, cisplatin, and high-dose methotrexate) remains the standard treatment for patients with osteosarcoma [9–11].

Mycophenolate mofetil is an immunosuppressive agent currently being used for the prophylaxis of organ rejection following transplantation [12, 13]. It is a prodrug of mycophenolic acid (MPA), a potent inhibitor of inosine monophosphate dehydrogenase (IMPDH) [14, 15]. In some cancer in which IMPDH is upregulated, MPA may possess anti-cancer activity [16]. In 2010, Fellenberg et al. reported the observed overexpression of IMPDH in metastatic and chemo-resistant osteosarcoma cell lines, and pharmacological inhibition of IMPDH could result in a reduction of cell viability and cell proliferation [17]. A recent in vitro study has revealed the anticancer activity of MPA against osteosarcoma cell lines, with the half maximal inhibitory concentration (IC_50_) of 0.46–7.30 μM across all tested cell lines [18]. The in vivo experiment has shown the inhibition of tumor growth and lung metastasis of osteosarcoma in mice treated with mycophenolate mofetil [18]. As a result, mycophenolate mofetil has been recently proposed to be a promising new drug candidate for the treatment of osteosarcoma [19].

With the therapeutic potential of mycophenolate mofetil against osteosarcoma in in vitro and animal experiments, further investigation in humans is warranted. Since mycophenolate mofetil has been widely used in humans for decades, particularly in organ transplant recipients, with a satisfactory safety profile, a phase I clinical trial aimed solely at determining the safety and tolerability of the drug may not be required. A single-arm phase II clinical trial is considered to be the first step in the translation of therapeutic potential of mycophenolate mofetil emerging from in vitro and animal studies into the clinical domain before the drug development process can move forward to a randomized-controlled, phase III clinical trial. The efficacy and safety of mycophenolate mofetil will be first assessed in patients with high-grade locally advanced or metastatic osteosarcoma in this proposed phase II clinical trial.

## Methods/design

### Study objectives

The primary objective of this study is to assess the anticancer activity of mycophenolate mofetil in patients with high-grade locally advanced or metastatic osteosarcoma, and the secondary objective is to assess the safety and tolerability of the drug. The present study also aims to explore the pharmacokinetics of mycophenolate mofetil, biomarkers, and the quality of life in patients with high-grade locally advanced or metastatic osteosarcoma.

### Study design

This prospective study is an open-label, single-arm, two-stage phase II clinical trial of mycophenolate mofetil orally administered at the dose of 5 g/day (or lower) for 4 cycles (28 days/cycle) or until disease progression or unacceptable toxicity. This two-stage phase II clinical trial plans to involve a total of 27 adolescent and adult patients with high-grade locally advanced or metastatic osteosarcoma. In Stage 1, 19 patients will be enrolled, and the trial will continue to Stage 2 and enroll additional 8 patients only if at least 3 patients in Stage 1 achieve the primary endpoint.

### Study setting

This trial will be conducted at multiple centers in Thailand, where the Faculty of Medicine, Chiang Mai University is the primary study site.

### Study endpoints

The primary endpoint of this trial is progression-free survival (PFS) (or disease control rate (DCR)) at 16 weeks. The secondary endpoints include PFS, overall survival (OS), overall response rate (ORR) (based on the RECIST criteria version 1.1) [20, 21], safety parameters (according to the Common Terminology Criteria for Adverse Events (CTCAE) version 5.0), pharmacokinetic (PK) parameters (i.e., plasma levels of MPA and its metabolites), biomarkers (consisting of lactate dehydrogenase and circulating tumor cells), pain score (using the Thai version of Brief Pain Inventory (BPI) [22]), and quality of life (using the EORTC QLQ-C30 questionnaire version 3.0 and the EORTC QLQ-LC13 questionnaire).

### Inclusion criteria

1. Evidence of histologically documented diagnosis of high-grade locally advanced or metastatic osteosarcoma, not amendable to surgery, radiation, or combined modality therapy with curative intent;
2. Measurable disease, determined within 28 days prior to enrollment;
3. Evidence of disease progression after treatment with, at least, one standard chemotherapy regimen for osteosarcoma or evidence of patient refusal for any further treatment with standard chemotherapy regimens for advanced disease;
4. ECOG performance status of ≤2, with an estimated life expectancy of > 3 months;
5. Age ≥ 13 years at the date of enrollment;
6. Adequate organ function, determined by laboratory tests within 14 days prior to enrollment; and
7. Informed consent obtained (or assent, when applicable).

### Exclusion criteria

1. History of another malignancy within 5 years prior to study entry, except curatively treated non-melanotic skin cancer or other solid tumors curatively treated with no evidence of disease for > 3 years;
2. Current treatment with another investigational agent and/or systemic anticancer therapy within 4 weeks prior to enrollment;
3. Surgery and/or radiotherapy for curative intent within 1 month prior to enrollment;
4. History of allergic reactions attributed to mycophenolate mofetil, MPA, allopurinol (including the presence of HLA-B*5801, indicating an increased risk of severe cutaneous adverse reactions to allopurinol), ivermectin, trimethoprim-sulfamethoxazole (or sulfa drugs), acyclovir or any ingredients of the drugs;
5. History of severe or uncontrolled medical conditions or laboratory abnormality;
6. Impaired renal function (with creatinine clearance of < 45 mL/min);
7. Known or suspected pregnancy or breastfeeding;
8. Any other conditions in which mycophenolate mofetil, allopurinol, ivermectin, trimethoprim-sulfamethoxazole, or acyclovir is contradicted;
9. Unable to swallow oral medications;
10. Major surgery within 4 weeks prior to study entry; or
11. Significantly altered mental status.

### Withdrawal criteria

1. More than 4 weeks of study drug interruption due to toxicity;
2. Disease progression or any significant deterioration in the health of the patient;
3. Unacceptable toxicity;
4. Occurrence of another illness which precludes further participation in this trial;
5. Significant protocol noncompliance;
6. Development of an illness or situation which would affect assessments of clinical status and study endpoints to a significant degree;
7. Pregnancy;
8. Patient lost to follow-up; or
9. Withdrawal of informed consent.

Patients who withdraw or are withdrawn prematurely will be replaced only if they discontinue during the first four weeks due to reasons other than toxicity.

### Interventions

Mycophenolate mofetil (CellCept®, Roche Laboratories Inc., Nutley, New Jersey) in 500 mg/tablet will be used in this trial. All adult patients (aged ≥18 years) will be treated with mycophenolate mofetil at the initial dose of 5 g/day, twice daily, while adolescent patients (aged 13–17 years) will be treated with mycophenolate mofetil at the initial dose of 3–5 g/day based on the patient’s body surface area (BSA) at enrollment (i.e., 5 g/day for those with a BSA of > 1.5 m^2^, 4 g/day for those with a BSA of 1.26 to 1.5 m^2^, and 3 g/day for those with a BSA of 1.0–1.25 m^2^). Self-administration of the study drug will take place on an outpatient basis (under parents’ supervision in some cases, as appropriate). Patients will return unused tablets at each follow-up visit; the unused tablets will be counted and recorded to determine compliance. One cycle is considered to be 4 weeks, and the next cycle of the treatment with mycophenolate mofetil will start only after safety assessments are done.

Allopurinol at the dose of 300–600 mg/day will be coadministered orally, twice daily, for blockage of the guanine salvage pathway [23]. All adult patients (aged ≥18 years) will be treated with allopurinol at the dose of 600 mg/day twice daily, while adolescent patients (aged 13–17 years) will be treated with allopurinol at the dose of 300 or 600 mg/day twice daily based on the patient’s body weight at enrollment (i.e., 600 mg/day for those with a body weight of ≥40 kg, and 300 mg/day for those with a body weight of < 40 kg). Evidence in kidney transplant recipients suggests that concurrent administration of mycophenolate mofetil with allopurinol is innocuous [24, 25].

Available palliative and supportive care for disease-related symptoms should be offered to all patients. Palliative radiotherapy is allowed for local pain control, provided that (1) the patient does not have progressive disease, (2) no more than 10% of the patient’s bone marrow is irradiated, and (3) the radiation field does not encompass a target lesion. Surgical resection of the disease is permitted after documentation of response. All the patients will be instructed not to take any other medications (including over-the-counter products) during study participation without prior consultation with the investigators.

### Dose modifications

Each patient will be closely monitored for toxicity, and the dose of mycophenolate mofetil may be adjusted according to individual patient tolerance at the discretion of the investigators (Table 1). The dose of mycophenolate mofetil may be reduced by 1–2 g/day or withheld for some Grade 3 or Grade 4 toxicities, whenever clinically needed (Tables 2). All dose-limiting toxicities will be managed following the standard protocol of a participating study site. The dose of mycophenolate mofetil for cycle 1 to cycle 4 of new patients may be reduced, at the discretion of the investigators, by 1–2 g/day, if more than 33% of the former patients experience a dose-limiting toxicity at that dose level and require dose de-escalation after treatment.

**Table 1:** Dose de-escalation schema of mycophenolate mofetil

Dose level	Dose of mycophenolate mofetil in adult patients
0 (initial dose)	5 g/day (5 tablets twice a day)
-1	4 g/day (4 tablets twice a day)
−2	3 g/day (3 tablets twice a day)
−3	2 g/day (2 tablets twice a day)
−4	1 g/day (1 tablet twice a day)

Note that the initial dose (dose level 0) of mycophenolate mofetil in pediatric patients is the dose that is adjusted based on the patients’ BSA at enrollment; dose level –x in pediatric patients will be the initial dose–x g/day

**Table 2:** Hematological and non-hematological criteria for suggested dose modification of mycophenolate mofetil

Toxicity^(a)^	Hold study treatment	Dose modification
*Hematological criteria*		
Grade 4 bone marrow hypocellular	No^(b)^	Decrease one dose level^(c)^
Grade 4 febrile neutropenia	No^(b)^	Decrease one dose level^(c)^
Grade 4 neutrophil count decreased	No^(b)^	Decrease one dose level^(c)^
≥ Grade 3 of other hematologic toxicities	No^(b)^	Decrease one dose level^(c)^
Sepsis & any Grade 3 infection	Yes until ≤ Grade 2^(d)^	Resume at one dose level lower^(c)^
Sepsis & any Grade 4 infection	Yes until ≤ Grade 2^(d)^	Resume at two dose level lower^(c)^
*Non-hematological criteria*		
Grade 3, except for: delayed puberty, growth suppression, breast atrophy, erectile dysfunction, diarrhea^(e)^, vomiting^(e)^, and AST/ALT increased or other biochemical laboratory abnormalities without any clinically significant sequelae	No^(b)^	Decrease one dose level^(c)^
Any Grade 4 toxicity	No^(b)^	Decrease two dose level^(c)^

^(a)^ If no recovery (until ≤ Grade 2) is noted after 7 days of dose modification of mycophenolate mofetil, that event will be considered as another toxicity requiring one more dose reduction; ^(b)^ Study treatment may be held whenever clinically needed (at the discretion of the PI and study team); ^(c)^ If more than 3 dose reductions are required, study treatment may be discontinued unless there is reasonable evidence of clinical benefit to justify continuation in the study; ^(d)^ If no recovery (until ≤ Grade 2) is noted after a 28-day delay, study treatment will be discontinued unless there is reasonable evidence of clinical benefit to justify continuation in the study; ^(e)^ Only if it occurs despite maximal medical treatment

### Participant recruitment

Potentially eligible patients will be identified by a treating physician, and approached by a study nurse. Accrual is expected to be complete within 2.5 years after trial initiation.

### Study procedures

Twelve visits are scheduled for this trial and a summary of study procedures for each visit is presented in Table 3. The expected duration of study participation is about 4–5 months. All patients must agree to use two reliable, effective contraceptive methods simultaneously for at least 7 days prior to the first dose of the study drug and for 6 weeks following the last dose of the study drug, unless absolute sexual abstinence is the chosen method of contraception. This is because mycophenolate mofetil is known to cause a high frequency of miscarriage (~ 50%) and severe birth defect in the unborn baby [26, 27].

**Table 3:** Scheduled visits and assessments

Procedures	Study Visits											
	SV	C_1_w_0_	C_1_w_1_	C_1_w_2_	C_1_w_4_	C_2_w_2_	C_2_w_4_	C_3_w_2_	C_3_w_4_	C_4_w_2_	C_4_w_4_	FV
Informed consent obtained	×											
Inclusion/exclusion criteria assessments	×											
History taking	×											
Physical examination	×	×	×	×	×	×	×	×	×	×	×	×
ECOG status	×											
Electrocardiogram	×											
Lab test for complete blood count	×			×	×	×	×	×	×	×	×	×
Lab test for fasting blood glucose	×											
Other lab tests^(a)^	×			×	×		×		×		×	×
Circulating tumor cells		×^(b)^	×^(b)^	×		×		×		×		
Biobank collection	×				×		×		×		×	×
Urine analysis^(c)^	×			×	×		×		×		×	×
Stool examination	×											×
Pharmacokinetic study^(d), (e)^		×	×									
Pain score & Quality of life		×			×		×		×		×	×
Review of concomitant medications	×	×	×	×	×	×	×	×	×	×	×	×
Monitoring for adverse events			×	×	×	×	×	×	×	×	×	×
Tumor assessment^(f)^	×						×				×	

SV = screening visit; C_x_w_y_ = cycle x week y; FV = final visit. ^(a)^ Lab tests include Na, K, Cl, HCO3, Ca, Mg, P, albumin, AST, ALT, ALP, total bilirubin, direct bilirubin, BUN, Cr, PT, PTT, INR, and LDH; ^(b)^ Blood obtained from the pharmacokinetic study will be used for measurement of circulating tumor cells on C_1_W_0_ and C_1_W_1_ so that no additional blood samples will be collected; ^(c)^ Urine analysis includes a urine pregnancy test for a female patient of childbearing age; ^(d)^ Blood samplings (at 0, 0.5, 1, 1.5, 2, 3, 4, 6, 8, 10, and 12 h); ^(e)^ Additional samplings for drug monitoring may be required when drug dosage is modified (after ≥7 days of drug administration with an adjusted dose level); ^(f)^ Tumor assessment by a computerized tomography (CT) scan (of the chest ± other organs, if required) or magnetic resonance imaging (MRI), done at baseline, 8 ± 1 weeks and 16 ± 1 weeks after initiation of study drug administration; in case of objective tumor response (complete response or partial response), confirmatory imaging studies will be performed at least 4 weeks after initial documentation of response

There are three drug regimens to be used for prophylaxis of possible infection during study participation: (1) ivermectin (12 mg/day) for 2 days before Cycle 1, Week 0 [28], (2) trimethoprim-sulfamethoxazole (160/800 mg/day) once daily from Cycle 1, Week 1, until discontinuation of mycophenolate mofetil [29], and (3) acyclovir (400 mg/day) twice daily from Cycle 1, Week 1, until discontinuation of mycophenolate mofetil [30]. No evidence of PK drug-drug interactions between any of these regimens and mycophenolate mofetil is reported [31].

Should mycophenolate mofetil prove beneficial to some patients (i.e., evidence of stable disease or partial response) at the end of the treatment period (on Week 16), the study treatment will be given to those patients in the extension period until one of the withdrawal criteria is met. Additional visits will be scheduled during the extension period (Table 4).

**Table 4:** Additional visits and assessments in the extension period

Procedures	Study Visits								
	C_5_w_2_	C_5_w_4_	C_6_w_2_	C_6_w_4_	C_7_w_2_	C_7_w_4_	C_x_w_2_	C_x_w_4_	FV
Physical examination	×	×	×	×	×	×	×	×	×
Lab test for complete blood count	×	×	×	×	×	×	×	×	×
Other lab tests^(a)^		×		×		×		×	×
Circulating tumor cells	×		×		×		×		
Biobank collection		×		×		×		×	×
Urine analysis^(b)^		×		×		×		×	×
PK study^(c)^									
Pain score & Quality of life		×		×		×		×	×
Review of concomitant medications	×	×	×	×	×	×	×	×	×
Monitoring for AEs	×	×	×	×	×	×	×	×	×
Tumor assessment^(d)^						×			

C_x_w_y_ = cycle x week y; FV = final visit. ^(a)^ Lab tests include Na, K, Cl, HCO3, Ca, Mg, P, albumin, AST, ALT, ALP, total bilirubin, direct bilirubin, BUN, Cr, PT, PTT, INR, and LDH; ^(b)^ Urine analysis includes a urine pregnancy test for a female patient of childbearing age; ^(c)^ Additional samplings for drug monitoring (at 0, 0.5, 1, 1.5, 2, 3, 4, 6, 8, 10, and 12 h) may be required when drug dosage is modified (after ≥7 days of drug administration with an adjusted dose level); ^(d)^ Tumor assessment by a CT scan (of the chest ± other organs, if required) or MRI, done at 12-week interval (±1 week); in case of objective tumor response (complete response or partial response), confirmatory imaging studies will be performed at least 4 weeks after initial documentation of response

The final visit will be scheduled in 4 weeks (±1 week) after the treatment with mycophenolate mofetil has stopped. After completion of the study, follow-up survival information will be collected by either clinical visit or telephone contact every 12 weeks (±1 week) until death.

### Sample size determination

The sample size was calculated according to the Simon’s minimax two-stage design, with PFS at 16 weeks as the primary endpoint. PFS is dichotomized according to whether PFS is ≤16 weeks (defined as disease control failure (DCF)) or > 16 weeks (defined as disease control success (DCS)). Therefore, treatment with mycophenolate mofetil will be considered successful if the 16-week radiological evaluation indicates stable disease, partial response, or complete response, as defined by the RECIST criteria version 1.1. Patients alive after 16 weeks without signs of progression will be regarded as DCS.

The sample size was calculated under a hypothesis of interest in which DCS at 16 weeks is ≥32% (*p*_1_ = 0.32, the minimal proportion of successes that makes the experimental treatment worth further studies) and a null hypothesis in which mycophenolate mofetil reaches a DCS at 16 weeks < 12% (*p*_0_ = 0.12, the proportion of successes that implies no clinically worthwhile activity of the experimental treatment). Setting *α* = 0.10 (where *α* represents the probability of failing to reject a treatment with response probability ≤*p*_0_) and *β* = 0.10 (where *β* represents the probability of rejecting a treatment with response rate ≥ *p*_1_), this condition requires 19 patients to be enrolled in Stage 1. Recruitment will continue to reach a total of 27 patients if the number of DCS among the first 19 patients is at least 3; otherwise, the trial will be stopped for inefficacy after analysis of 19 patients. As per the study protocol, mycophenolate mofetil will be deemed promising and worth further studies if at least 6 DCS is observed at the end of the study.

### Stopping rules

In serious diseases (like high-grade locally advanced or metastatic osteosarcoma), the trial may need to stop prematurely if there is evidence of inefficacy [32]. Thus, this two-stage phase II clinical trial with a futility stopping based on Simon minimax criterion will be prematurely stopped if there is fewer than 3 DCS among the first 19 patients treated with mycophenolate mofetil.

### Data management

This trial uses Research Electronic Data Capture (REDCap), a secure web-based application for managing clinical trial databases. The study essential documents will be retained, as the investigators’ responsibility, for a minimum of 15 years after completion or discontinuation of the study or for a longer period if required.

### Data monitoring

In this open-label, single-arm phase II clinical trial, Data Monitoring Committee (DMC) is not needed because of a small number of patients involved (*n* = 27) and that the investigators can closely monitor the safety of all individual patients taking a known drug with a known dose (open-label). This allows the investigators to take appropriate action in time.

### Statistical analysis plan

All patients who receive at least one dose of mycophenolate mofetil will be included in an intention-to-treat analysis. Patients’ baseline demographics will be summarized using descriptive statistics. Study drug administration will be described in terms of the total number of cycles (or days) administered, the median (range) of cycles (or days) administered, dose intensity, and reasons for deviations from planned therapy. Adverse events will be collected after the patient has taken the first dose of mycophenolate mofetil.

The PFS at 16 weeks or DCR (the primary endpoint) will be measured as a binary variable: DCS or DCF. The PFS and OS will be summarized using the Kaplan-Meier method and displayed graphically as appropriate. For patients lost to follow-up, they will be censored at the date of the last follow-up visit. Median PFS and OS (and their corresponding 95% confidence intervals) will also be provided.

All statistical analysis will be executed using IBM SPSS Statistics for Windows, version 22.0, with a *p* value of less than 0.05 considered to indicate statistical significance. It is possible that the decision rule of this single-arm, two-stage phase II clinical trial may need to be adapted from the original plan [33]. If the attained sample size at the end of this study is different from the one that is initially planned due to any reason, a statistical testing will be conducted by calculating the *p* value with the sample size at the stopping stage conditioned on the observed value [34, 35]. The Jennison-Turnbull confidence interval may be used by treating the observed sample size at the stopping stage like the planned sample size [36].

### Ethical approval and trial status

This phase II clinical trial is to be conducted in accordance with applicable international standards of Good Clinical Practice (ICH E6R2 2016) and Declaration of Helsinki 2013, as well as applicable institutional research policies and procedures. The Research Ethics Committee of the Faculty of Medicine, Chiang Mai University approved this study protocol and related documents on June 28, 2019. This trial was prospectively registered on June 30, 2019 (registration number: TCTR20190701001). Approval will be obtained from the local ethics committees (i.e., The Khon Kaen University Ethics Committee in human research; The Research Ethics Committee, Faculty of Medicine, Prince of Songkla University; and The Research Ethics Committee of Lerdsin Hospital, Department of Medical Services, Ministry of Public Health) before starting patient accrual at each institution. Any modifications to the protocol and related documents will be submitted to ethics committees for approval of such amendments prior to implementation.

## Discussion

This is the first prospective phase II clinical trial which is designed to assess the efficacy and safety of mycophenolate mofetil in patients with high-grade locally advanced or metastatic osteosarcoma. The results of this trial will reveal the therapeutic potential of mycophenolate mofetil against osteosarcoma in humans for the first time. To our knowledge, there have been no new drugs (either chemotherapeutic, molecule-targeted, or immunotherapeutic agents) found to be active against osteosarcoma for decades notwithstanding that there is a substantial need for the discovery and development of novel agents so as to improve survival outcome of patients particularly for whom the prognosis is unfavorable [37].

### Justification for the study design and study endpoints

In phase II osteosarcoma trials, a single-arm design may be preferred to a randomized-controlled design owing to the fact that osteosarcoma is a rare disease, with a limited number of patients available to be enrolled in early-phase clinical trials with a specific and strict set of eligibility criteria [38]. Based on a systematic review of past experience, most phase II osteosarcoma trials are conducted using a single-arm, two-stage design [39]. A single-arm design is considered appropriate for the evaluation of monotherapy when a well-defined historical control database is available, while a key limitation of this approach is that any changes in patient management over time may shift the expected outcome (e.g., PFS) above the historical benchmark [40]. In the case of osteosarcoma, the standard treatment for newly diagnosed and recurrent disease has not substantially been changed over the past three decades, so that the historical benchmark for the outcome of patients could be reliably used [41].

One of the major characteristics of osteosarcoma is that tumor volume shrinkage, determined by radiographic imaging, may not reflect the efficacy of an anticancer agent at the cellular level since tumor tissue is customarily substituted by the calcified matrix [42]. Even if the treatment is effective, the calcified matrix may still prevent volume reduction of the tumor [43]. Hence, objective radiographic responses in osteosarcoma are rarely observed, even with proven complete necrosis in the tumor after neoadjuvant chemotherapy [44]. In addition, the chance of spontaneous stability in advanced osteosarcoma is estimated to be extremely low [45]. Therefore, it may be reasonable to regard ‘stable disease’, rather than only ‘complete response’ and ‘partial response’, at a pre-specified, justified period as an appropriate surrogate endpoint in this specific group of population [39]. This is in agreement with the recent analysis of seven phase II osteosarcoma trials, where PFS > 4 months (defined as DCS) is used as a primary endpoint [44]. It is, thus, reasonable to consider PFS at 16 weeks (or disease stabilization at 16 weeks) as a success in this ESMMO trial setting where a decrease of tumor volume is not expected.

### Justification for involving adolescent patients in the early-phase clinical trial

Enrollment of adolescent patients (aged > 13 years) in this trial with a specific and strict set of eligibility criteria is justified by the fact that osteosarcoma is a rare disease by which the population affected is predominantly teenagers and young adults [2, 4]. According to the guidance of ‘Considerations for the inclusion of adolescent patients in adult oncology clinical trials’ (84 Federal Register 49 (March 13, 2019)), adolescent patients can be enrolled simultaneously with adults in an early-phase clinical trial, provided that they have cancer that is relapsed after or refractory to standard chemotherapy with no curative options or for which no standard therapies with curative intent exist. This is consistent with a systematic review of phase II osteosarcoma trials, in which two thirds (67/99) of the trials between 2003 and 2016 enrolled both adult and pediatric patients simultaneously [39]. In most cases, the toxicity profiles of anti-osteosarcoma chemotherapeutic agents in adolescent patients are similar to those experienced by adult patients [46]. Consequently, testing the efficacy and safety of mycophenolate mofetil in both adolescent and adult patients with high-grade locally advanced or metastatic osteosarcoma in this proposed phase II clinical trial could be ethically justified.

### Rationale for the experimental dose of mycophenolate mofetil

The initial dose of mycophenolate mofetil at 5 g/day which is planned to be used in this phase II clinical trial is based on the pharmacologically active dose in mice, from which human equivalent dose is then calculated, as well as clinically experienced doses in humans. Tumor growth inhibition was evident in mice treated with mycophenolate mofetil at 200 mg/kg/day [18], so mycophenolate mofetil at the dose of 1 g/day or more is expected to be pharmacologically active in humans. In clinical practice, long-term treatment with mycophenolate mofetil at the dose of 2 to 3 g/day has a satisfactory safety profile in organ transplant recipients [47, 48]. In addition, it is evident that mycophenolate mofetil at the dose of 4 to 5 g/day is fairly tolerated [49, 50]. The most severe dose-limiting adverse effects of mycophenolate mofetil are gastrointestinal disturbances (e.g., diarrhea, abdominal pain, nausea, and vomiting) and those related to immunosuppression (e.g., leukopenia and infection) ([51]; Keown 1996). For chemotherapeutic agents, it is generally assumed that higher doses of the drug would produce more efficacy against cancer [52]. Considering the evidence available to date, the oral dose of 5 g/day was chosen to be the experimental dose of mycophenolate mofetil in this trial. The rationale behind this chosen dose (5 g/day) of mycophenolate mofetil, rather than lower ones (2 or 3 g/day), is to reduce the likelihood of sub-therapeutic exposure to the anticancer drug, while the toxicity of the drug, if any, can be closely monitored and managed. The initial dose of mycophenolate mofetil given to new patients may be reduced, at the discretion of the investigators, according to clinical circumstances observed in the former patients; the trial protocol will be amended if the initial dose is subject to dose modification.

### Sample size justification

In this trial, the sample size determination is based on the rigorous evidence available to date so as to minimize the false positive rate owing to a single-arm design [53]. An under-estimation of *p*_0_ induces an increased type I error, while an over-estimation of *p*_1_ implies a loss of power [54]. In the retrospective analysis of the outcome of patients with recurrent/refractory osteosarcoma in seven phase II clinical trials conducted between 1997 and 2007, the DCS at 4 months is 12% (95% CI: 6 to 19%) [44]. The mean DCS probability of 12% is, thus, chosen to be the baseline response rate expected to be observed should mycophenolate mofetil be ineffective in this trial (*p*_0_ = 0.12). The effect size (*p*_1_-*p*_0_) that the experimental drug would still be of interest for further development is set at 20% (*p*_1_ = 0.32) [55]. In inoperable osteosarcoma settings, a median time to progress is around 1.8 months, with a median survival of around 6 months and 2-year survival of < 2% [45, 56, 57]. Therefore, the proportion of DCS of 32% can be considered appropriate to reflect the efficacy of mycophenolate mofetil whether or not it is worth further development [58].

In this trial, the minimax design is chosen to minimize the maximum sample size under the null hypothesis [55]. This is because the difference in expected sample sizes in this scenario is trivial as well as the patient accrual rate may be low. Furthermore, patients with high-grade locally advanced or metastatic osteosarcoma may be heterogeneous, so it is reasonable to assume that the patients entering early in the trial may not be representative of eligible population [55]. A small first stage may not be desirable, and the minimax design is preferable to the optimal design accordingly.

## Data Availability

Not applicable.

## Abbreviations

BPI: Brief pain inventory
BSA: Body surface area
CTCAE: Common terminology criteria for adverse events
DCF: Disease control failure
DCR: Disease control rate
DCS: Disease control success
DMC: Data monitoring committee
ECOG: Eastern cooperative oncology group
EORTC: European organization for research and treatment of cancer
ESMMO: Efficacy and safety of mycophenolate mofetil in patients with high-grade locally advanced or metastatic osteosarcoma
IC_50_: Half maximal inhibitory concentration
IMPDH: Inosine monophosphate dehydrogenase
MPA: Mycophenolic acid
ORR: Overall response rate
OS: Overall survival
PFS: Progression-free survival
PK: Pharmacokinetic
QLQ-C30: Quality of life of cancer patients
RECIST: Response evaluation criteria in solid tumors
REDCap: Research electronic data capture
